# Efficacy of vitamin D supplementation in gestational diabetes mellitus: Systematic review and meta-analysis of randomized trials

**DOI:** 10.1371/journal.pone.0213006

**Published:** 2019-03-22

**Authors:** Meline Rossetto Kron Rodrigues, Silvana Andréa Molina Lima, Glaucia Maria Ferreira da Silvia Mazeto, Iracema Mattos Paranhos Calderon, Claudia Garcia Magalhães, Guilherme Augusto Rago Ferraz, Ana Claúdia Molina, Roberto Antônio de Araújo Costa, Vania dos Santos Nunes Nogueira, Marilza Vieira Cunha Rudge

**Affiliations:** 1 Department of Gynecology and Obstetrics, São Paulo State University (UNESP), Medical School, Botucatu, São Paulo, Brazil; 2 Nursing Department, São Paulo State University (UNESP), Medical School, Botucatu, São Paulo, Brazil; 3 Department of Internal Medicine, São Paulo State University (UNESP), Medical School, Botucatu, São Paulo, Brazil; 4 Municipal Authority of Botucatu, São Paulo, Brazil; Bradford Institute for Health Research, UNITED KINGDOM

## Abstract

**Background:**

Trials have examined on the benefits of vitamin D supplementation in pregnant women.

**Objective:**

This review aimed to evaluate whether oral vitamin D supplements, when given to pregnant women with gestational diabetes mellitus (GDM), would improve maternal and neonatal outcomes, compared with no treatment or placebo.

**Method:**

We performed a systematic review following Cochrane methodology, and randomized trials were included where pregnant women with GDM received vitamin D supplementation versus placebo/no treatment or vitamin D and calcium versus placebo/no treatment. Primary outcomes were preeclampsia, preterm birth, cesarean delivery, gestational hypertension, and adverse events related to vitamin D supplementation. The search strategies were applied to the following databases: MEDLINE, Embase, LILACS, and CENTRAL. Similar outcomes in at least two trials were plotted using Review Manager 5.3 software. The quality of evidence was generated according to the Grading of Recommendations Assessment, Development, and Evaluation (GRADE).

**Results:**

The total of 1224 references were identified, eleven trials were potentially eligible, and six were included in this review (totaling 456 women). The meta-analysis of frequency of cesarean deliveries did not show significant differences between groups, none of the trials evaluated the remaining primary outcomes. For secondary outcomes, our results suggest that vitamin D supplementation in pregnant women with GDM may reduce newborn complications such as hyperbilirubinemia, polyhydramnios (RR: 0.40, 95% CI: 0.23 to 0.68; RR: 0.17, 95% CI: 0.03 to 0.89; respectively), and the need for maternal or infant hospitalization (RR: 0.13; 95% CI: 0.02 to 0.98; RR: 0.40, 95% CI: 0.23 to 0.69). However, the evidence was of low or very low quality.

**Conclusion:**

We did not find moderate or high quality evidence indicating that vitamin D supplementation, when compared with placebo, improves glucose metabolism, adverse maternal and neonatal outcomes related to GDM in pregnant women.

## Introduction

GDM is associated with maternal and neonatal risks [1]. A large, multinational cohort study, Hyperglycemia and Adverse Pregnancy Outcome study [2], demonstrated that GDM or obesity alone, compared with normoglycemic pregnant controls, had significantly greater odds of low birth weight, newborn percent body fat, primary cesarean delivery, and preeclampsia. In addition, the risk of adverse maternal, fetal, and neonatal outcomes continuously increased as a function of maternal glycemia at 24–28 weeks, even within ranges previously considered normal [2]. In addition, a systematic review showed a positive association between maternal hyperglycemia and caesarean section, induction of labor, large for gestational age, macrosomia, and shoulder dystocia [3].

Vitamin D is a group of fat-soluble secosteroids predominantly found in fish-liver oils, fatty fish, mushrooms, egg yolks, and liver. Furthermore, vitamin D can also be produced in the body in the presence of sunlight [4]. Its two physiological active forms are vitamin D_3_ (also known as cholecalciferol) and vitamin D_2_ (ergocalciferol). In response to parathyroid hormone, both forms are first hydroxylated in the liver to 25-hydroxyvitamin D (25(OH)D or calcidiol), and sequentially converted to 1,25-dihydroxyvitamin D (calcitriol) in the kidneys. Vitamin D_3_ is three times more effective than vitamin D_2_ in increasing vitamin D concentrations and maintaining those levels for a longer period of time [5].

The most common function of vitamin D is the maintenance of calcium homeostasis and bone integrity. However, besides bone and parathyroid glands, there are other sites of vitamin D action, which includes the skin, intestines, immune system, and pancreas. It is known that vitamin D has immunomodulatory and anti-inflammatory effects [6], and these effects aids glucose metabolism by regulating the release of insulin according to levels of glucose [7–10].

This effect of vitamin D explains the association between maternal vitamin D deficiency in early pregnancy and the elevated risk for GDM, and it has been demonstrated in some observational studies [11]. A case-control study involving 1280 women with GDM and 3438 controls evaluated the association of 25(OH)D concentrations with risk of GDM. After adjusting for confounding factors, women with low concentrations (< 50.0 nmol/L) of 25(OH)D displayed a significantly increased risk of GDM and adverse pregnancy outcomes (e.g., anemia, macrosomia, abnormal amniotic fluid, and stillbirth or miscarriage) [12].

A cohort study conducted in Brazil evaluated the effect of vitamin D deficiency on neonatal outcomes of pregnant women with GDM. The authors identified that newborns of women with vitamin D deficiency had a significantly higher incidence of hospitalization in critical care units, hypoglycemia, and small size for gestational age. The incidence of prematurity, jaundice, and dystocia of the shoulder were not statistically significant between groups [13].

As vitamin D supplementation can be easily administered without apparent serious adverse events, and vitamin D deficiency is frequent among pregnant women, studies have examined on vitamin D supplementation in pregnant women with GDM [13,14]. Li et al. [14] evaluated the effect of vitamin D_3_-supplemented yogurt on glucose metabolism and lipid concentrations in pregnant women with GDM. After 16 weeks of intervention, both fasting plasma glucose and lipid levels were markedly lower in the supplementation group than control participants.

Thus, under the hypothesis that vitamin D supplementation during pregnancy protects pregnant women against adverse outcomes related to GDM, this review aimed to evaluate whether oral supplements with vitamin D given to pregnant women with GDM would improve maternal and neonatal outcomes, compared to no treatment or placebo.

## Materials and methods

### Protocol and registration

We performed a systematic review following Cochrane methodology [15] and reported according to the Preferred Reporting Items for Systematic Reviews and Meta-Analyses guidelines [16]. Its protocol was registered in the International Prospective Register of Systematic Reviews database, accessible under the protocol number CRD42016021971.

### Eligibility criteria

We included randomized controlled trials (RCT), which adopted the Patient Intervention Comparison Outcome structure.

### Patients

We were exclusively interested in pregnant women diagnosed with GDM. We considered GDM as diabetes diagnosed in the second or third trimester of pregnancy by fasting plasma glucose levels or oral glucose tolerance test (50 and 100 g) at 24-28th week of gestation, and according to either the American Diabetes Association, World Health Organization, National Institute of Health, or International Association of the Diabetes and Pregnancy Study Groups.

### Intervention

We considered vitamin D supplementation as vitamin D_3_ (cholecalciferol) regardless of dose, duration, or time of initiation. This supplementation could be alone or in combination with other vitamins and minerals (e.g., calcium).

### Comparison

We considered control groups as those receiving no intervention or placebo.

### Outcomes

Primary maternal outcomes were preeclampsia, preterm birth (i.e., gestation less than 37 weeks), cesarean delivery, gestational hypertension and adverse events.

The neonatal primary outcomes were stillbirth, neonatal death (within 28 days postpartum), low birth weight (< 2,500 g), Apgar less than 7 at 5 minutes, and neonatal infection (respiratory infections within 28 days after delivery).

We considered secondary maternal outcomes as decreased fasting glucose, glycated hemoglobin, lipid profile (total cholesterol, High Density Lipoprotein (HDL) cholesterol, Low Density Lipoprotein (LDL) cholesterol, triglycerides), homeostasis model of assessment (HOMA) for insulin resistance index (HOMA-IR), HOMA-B (b cell function), serum concentration of vitamin D (25-hydroxyvitamin D in nmol/L), serum calcium concentration, change in BMI, newborn complications (e.g., polyhydramnios and hyperbilirubinemia), insulin use after supplementation, maternal hospitalizations after supplementation, frequency of newborn hospitalization, and macrosomia.

### Exclusion criteria

We excluded trials of pregnant women without GDM, with calcium metabolic disorder, known diabetes prior to pregnancy, and diabetes diagnosed in the first trimester of gestation.

### Search strategy

The following electronic databases were consulted: MEDLINE (via PubMed; 1968 to September 12, 2017), EMBASE (1989 to September 12, 2017), CENTRAL (Cochrane Collaboration Controlled Register; 1972 to September 2017), and LILACS (Latin American and Caribbean Literature on Health Sciences; 1982 to September 12, 2017) on the Virtual Health Library website. Information on ongoing RCTs was consulted through the Clinical Trials website of the National Institute of Health (http://clinicaltrials.gov) and through the Brazilian Registry of Clinical Trials-ReBEC (http://www.ensiosclinicos.gov.br).

The basic research strategy was developed for PubMed and modified as required for other databases (S1 Appendix). We used the health descriptors available in Descriptors in Health Sciences and Medical Subject Heading. The basic research strategy included “Diabetes, Gestational,” “Diabetes Mellitus, Type 2”, “Pregnancy”, “Vitamin D”, “Cholecalciferol”, and “Vitamin D Deficiency”. There was no language restriction. References to selected articles, including relevant review articles, were reviewed to identify all relevant studies. A manual search of references of RCTs was carried out in relevant journals and congresses in the area.

### Selection of studies

For this review, two researchers (MRKR and SAML) independently reviewed the eligibility of the titles and abstracts. The studies potentially eligible for inclusion were selected for full reading and subsequently assessed for compliance with the Patient Intervention Comparison Outcome structure. Disagreements regarding the selection of articles were resolved by discussion with a third researcher (VSNN).

### Data extraction

Two researchers (MRKR and SAML) independently extracted relevant data (participants, specific vitamin D intervention, and outcome characteristics) from each full-text article, using a standardized form based on the Cochrane Handbook [15]. The selection was compared for accuracy, and any discrepancies were resolved by consensus or discussion with another researcher (VSNN). If necessary, the corresponding authors of the original studies were contacted to obtain missing information. In cases of duplicate publications or multiple reports from the primary study, data extraction was optimized using the best information available for all items.

### Evaluation of risk of bias

Two investigators (MRKR and SAML) independently assessed the risk of bias of each eligible RCT. Any discrepancies were resolved by consensus or discussion with another investigator (VSNN). The Cochrane Collaboration tool for risk assessment of bias in RCTs was used, which includes seven criteria described in the Cochrane Reviewers’ Manual [15].

### Data synthesis and analysis

Similar outcomes in at least two trials were plotted using Review Manager 5.3 software. Continuous data were expressed as mean difference (MD) and standard deviations, and the difference of means with 95% confidence interval (CI) was used as an estimate of the intervention effect. For dichotomous data, the relative risk (RR) was also calculated with 95% CI. The random effect model was used, while the inverse variance method was used to weigh the effect estimates between included trials. The inconsistency of variance between the results of the included trials was ascertained by the Higgins inconsistency test (I^2^), [15] where I^2^ < 25% indicated low probability, I^2^ = 50% moderate probability and, I^2^ > 75% indicated a high probability of heterogeneity. If vitamin D levels were given in ng/mL, the values were converted using the following formula: 1 ng/mL = 2.5 nmol/L.

### Quality of evidence

The quality of evidence of the intervention effect estimates for the outcomes that could be plotted was generated according to GRADE (Grading of Recommendations Assessment, Development, and Evaluation) [17].

## Results

### Selected articles

**Fig 1:**
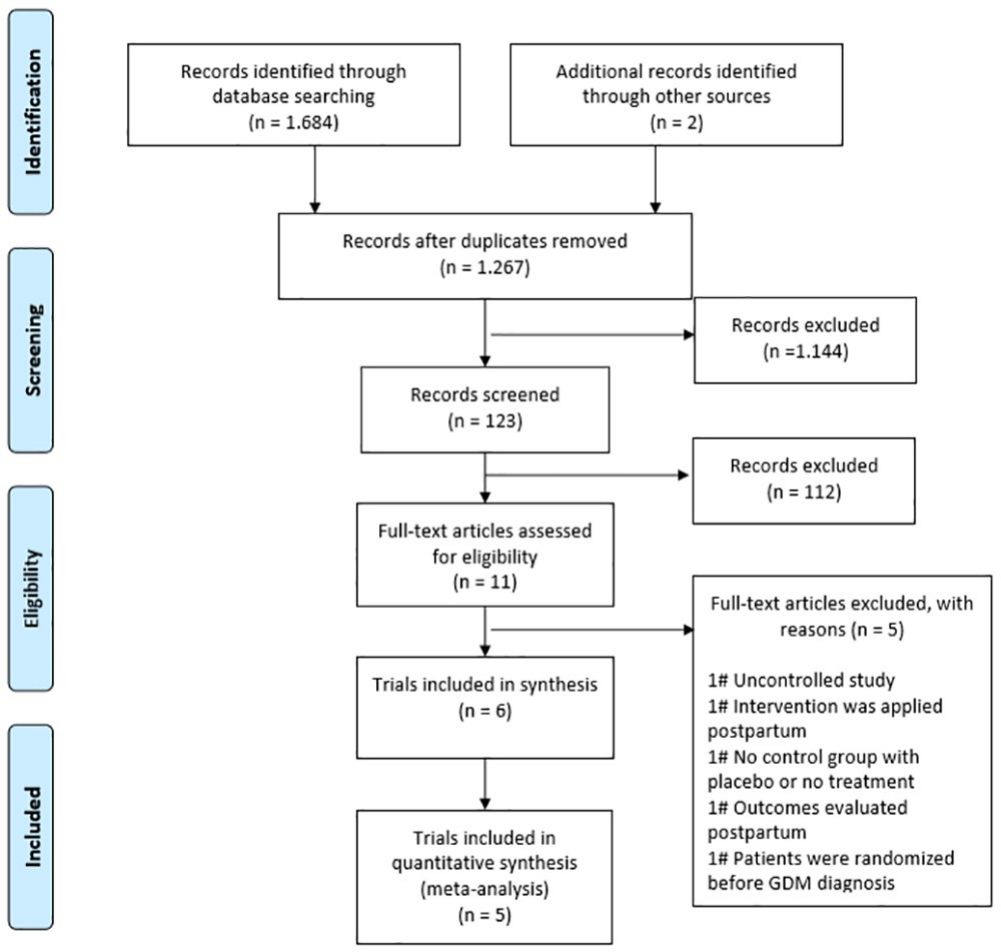
Flowchart for identifying eligible studies.

After searching the electronic health databases, 1224 references were identified (Fig 1). Eleven articles were potentially eligible for inclusion in this review and were therefore read in full. After reading in full, six studies met the inclusion criteria and were included [18–22]. One trial demonstrated two outcomes of interest for this systematic review (fasting glucose and calcium concentration), but the data could not be plotted in the meta-analysis, as they were not expressed in MD and standard deviation, even after contacting the authors [22].

Five studies were excluded. One study was excluded because it was uncontrolled [23]; in one study vitamin D supplementation was applied at 6 to 48 months postpartum [24]; in one study the authors randomized the patients to different doses of vitamin D (i.e., there was no control group with placebo or no treatment) [25]; one study was excluded because the selected outcomes (glycated hemoglobin, serum concentration of vitamin D and serum calcium concentration) were evaluated only postpartum [26]; and the last exclusion occurred because the patients were randomized before GDM diagnosis [27].

### Description of the studies

Six RCTs were included, totaling 456 pregnant women with GDM diagnosed in the second or third trimester of pregnancy. GDM diagnosis was according to criteria by the American Diabetes Association. The trials were published between 2013 and 2016; four were developed in Iran [18–21] and two in China [14, 22].

Tables 1 and 2 show the baseline characteristics and eligibility criteria of the included trials. In all trials, intervention and controls groups were similar in maternal ages, maternal height, weight at the beginning of the intervention, BMI, and concentration of 25(OH)D. In all trials, the gestational age at onset of intervention was the third trimester of pregnancy, and the status of 25(OH)D at baseline visit was not different between groups, with a mean level of approximately 20 ng/mL.

In the four Iranian trials, the period of treatment was six weeks, Li et al. [14] was 16 weeks, and Zhang et al. [22] was until delivery.

In the four Iranian trials, [18–21] the intervention participants were prescribed 50,000 IU of vitamin D_3_ every 2 weeks. In Zhang et al. (2016), [22] there was more than one intervention group, and the maximum dose of vitamin D_3_ was 50,000 IU every 2 weeks. In Li et al. (2016), [14] the vitamin D supplementation occurred using a vitamin D_3_-supplemented yogurt drink, providing 1000 UI daily. In all trials, the control groups were given a placebo. Two of the Iranian trials also administered calcium carbonate (1000 mg daily) to the intervention groups [19, 21].

**Table 1:** Characteristics of studies assessed at baseline.

	Sample size (n)	Age at baseline (year)	Maternal height (cm)	Maternal weight (kg)	BMI(kg/m2)	25 OH Vit D (ng/mL)	Gestational Age (weeks)
**Asemi, 2014**	G1:28 G2:28	G1:28.7±6.0 G2:30.8±6.6 P = NI	G1:158.1±4.6 G2: 159.9±4.4 P = NI	G1:73.6±13.0 G2:78.2±13.6 P = NI	G1:29.4±4.6 G2: 30.5±4.6 P = NI	G1:17.24±11.27* G2: 19.62±13.72* P = NI	G1:NI G2:NI Mean GI: 25.6±1.3 for both groups
**Asemi, 2015**	G1:25 G2:25	G1:31.1±5.5 G2:30.8±6.2 P = 0.88	G1:160.7±6.7 G2:159.6±4.5 P = 0.53	G1:79.0± 9.7 G2:77.8±12.9 P = 0.73	G1:30.7±3.9 G2: 30.5±4.4 P = 0.86	G1:18.9 ±14.5 G2:20.9 ± 14.3 P = 0.63	G1:25.3±1.2 G2:25.8 ± 1.3 P = 0.17
**Asemi, 201**	G1:27 G2:27	G1:31.7±5.6 G2:31.8±6.6 P = 0.96	G1:160.7±6.8 G2:159.4± 4.2 P = 0.41	G1:79.3±9.5 G2:78.3±13.4 P = 0.74	G1:30.9±4.5 G2:30.7±4.5 P = 0.89	G1: 20.44±14.31 G2: 20.41±13.43 P = NI	G1:NI G2:NI P = NI
**Karamali, 2015**	G1:30 G2:30	G1:28.7±6.1 G2:31.6±6.3 P = 0.06	G1:158.2± 6.1 G2:159.9±4.3 P = 0.14	G1:73.7 ±12.8 G2:78.1±13.4 P = 0.18	G1:29.4.7 G2:30.5±4.5 P = 0.36	G1:17.3 (±10.9) G2:20.8 (±14.4) P = 0.29	G1:25.5(±1.2) G2:25.6 (±1.3) P = 0.60
**Li, 2016**	G1:48 G2:49	G1: 29.0±5.3 G2: 28.3±4.1 P = 0.45	G1:1.661±0.07 G2:1.648±0.05	G1: 67.9±7.1 G2: 69.3±6.7 P = 0.61	G1:NI G2:NI	G1:16.8±4.6 G2:16.2±3.4 P = 0.02	G1: 14.5±1.1 G2: 14.2±1.2 P = 0.54
**Zhang, 2015**	G1:37 G2:20	G1: 30.1±4.5 G2:29.8±4.7 P = 0.87	G1:159.1±5.1 G2:160.1±5.1 P = 0.62	G1:78.8±12.1 G2:79.1±10.1 P = 0.76	G1:30.6±4.1 G2:31.1±3.9 P = 0.63	G1:NI G2:NI	G1:NI G2:NI

G1: Intervention, G2: Control, NI: Not informed

* values converted by 1ng / mL = 2.5 nmol

**Table 2:** Characteristics and information of trials evaluated in this systematic review.

	First author, year published					
	Asemi, 2014 [30]	Asemi, 2015 [31]	Asemi, 2013 [29]	Karamali, 2015 [32]	Li, 2016[14]	Zhang, 2016[22]
**Study location**	Kashan, Iran	Kashan, Iran	Kashan, Iran	Arak, Iran	Cangzhou, China	Shanghai, China
**Source of funding**	Kashan University of Medical Sciences	NI	Kashan University of Medical Sciences	Arak University of Medical Sciences	NI	NI
**No. of participants**	56	45	54	60	103	133
**Age (y), mean (SD)**	G1: 28.7±6.0 G2: 30.8±6.6 P = NI	G1: 31.1 ± 5.5 G2: 30.8 ± 6.2 P = 0.88	G1: 31.7 ± 5.6 G2: 31.8 ± 6.6 P = 0.96	G1:28.7 (±6.1) G2:31.6(±6.3) P = 0.06	G1: 29.0±5.3 G2: 28.3±4.1 P = 0.45	G1: 30.1±4.5 G2:29.8±4.7 P = 0.87
**Treatment duration (wk)**	6	6	6	6	16	Until the delivery
**Inclusion criteria**	Pregnant women aged 18–40 years with diagnosis of GDM (by ADA*).	Pregnant women of the firstborn of 18 and 40 years diagnosed with GDM (by ADA **)	Pregnant women aged 18–40 years with diagnosis of GDM (by ADA**).	Pregnant women aged 18–40 years with diagnosis of GDM (by ADA*).	Pregnant women aged 24–32 years who were carrying singleton pregnancy and diagnosed with GDM (by ADA*) at the onset of their second trimester (13 weeks).	Pregnant women with GDM during weeks 24-28 of pregnancy
**Exclusion Criteria**	Pregnant women with premature rupture of the placenta, placenta detachment, preeclampsia, eclampsia, chronic hypertension, hypothyroidism, urinary tract infection, renal diseases, liver diseases, stressful living conditions, smokers, use of estrogen therapy were not included in the study. We excluded those who started insulin therapy during the intervention.	Pregnant women with premature rupture of the placenta, placenta detachment, pre-eclampsia, hypothyroidism, urinary tract infection, renal diseases, liver diseases, smokers, and estrogen therapy were not included in the study. We excluded those who started insulin therapy during the intervention.	Pregnant women with premature membrane rupture, placenta detachment, preeclampsia, eclampsia, hypothyroidism, urinary tract infection, kidney disease, liver disease, smokers, and estrogen therapy were not included in the study. We excluded those who started therapy with during the intervention.	Pregnant women with premature membrane rupture, twin pregnancy, diagnosis of congenital fetal abnormalities, use of illicit drugs, calcium and / or vitamin D supplementation since the last menstrual period, insulin deficient, smokers and pregnant women with renal diseases were not included in the study.	Pregnant women with history of diabetes, pre-eclampsia, eclampsia, hypo- and hyperthyroidism, urinary tract infection, multiparity, maternal hypertension, liver, kidney or renal disease, those requiring insulin therapy during the study and those who consumed any type of vitamin D supplements (including yogurt drink supplemented with vitamin D) during the previous 6 months	Pregnant women with diabetes or GDM treated with insulin, thyroid or parathyroid disorders, polycystic ovary disease prior to pregnancy, a body mass index (BMI) of >30 kg/m2 prior to pregnancy and women who had received vitamin D supplementation in the 6 months that preceded the trial.
**Treatment group**	G1: Calcium carbonate (1,000 mg) daily and vitamin D3 capsule (50,000U) twice during treatment, on the 1st day and on the 21st day of intervention (n = 28) G2: Daily Calcium Placebo and twice placebo of Vitamin D3 during treatment, on the 1st day and on the 21st day of intervention (n = 28)	G1: Vitamin D3 (50,000 U) twice during treatment, on the 1st day and on the 21st day of intervention (n = 25). G2: Vitamin D placebo twice during treatment, on day 1 and day 21 (n = 25).	G1: Vitamin D3 (50,000 U) twice during treatment, on the 1st day and on the 21st day of intervention (n = 27). G2: Vitamin D placebo twice during treatment, on day 1 and day 21 (n = 27).	G1: Calcium carbonate (1,000 mg) daily and vitamin D3 capsule (50,000U) twice during treatment, on the 1st day and on the 21st day of intervention (n = 30). G2: Daily Calcium Placebo and twice placebo of Vitamin D3 during treatment, on the 1st day and on the 21st day of intervention (n = 30).	G1: Consume 2 servings (100 g per serving) of either plain yogurt VDY drink (‘PY’ supplemented with 500 IU vitamin D3), with one serving at breakfast and the other one at dinner, on a daily basis for a period of 16 weeks (n = 52) G2: Consume 2 servings (100 g per serving) of either plain yogurt (PY) drink (‘PY’ without any vitamin D 3 supplement) with one serving at breakfast and the other one at dinner, on a daily basis for a period of 16 weeks (n = 51)	G1: The low dosage group (n = 38) received the daily recommended intake of 200 IU vitamin D (calciferol) daily, the medium dosage group (n = 38) received 50,000 IU monthly (2,000 IU daily for 25 days) and the high dosage group (n = 37) received 50,000 IU every 2 weeks (4,000 IU daily for 12.5 days). G2:The control group (n = 20) received a placebo (sucrose; one granule/day),
**Primary endpoint**	Vitamina D (nmol / L) Calcium (mol / L) FPG (mol / L) Insulin (mol / L) HOMA-IR -HOMA-B QUICKI Total cholesterol (mmol / L)—Triacylglycerol (mmol /L) LDL cholesterol (mmol / l) HDL cholesterol (mmol / l) Total: HDL-cholesterol hs-CRP (ng / ml) NO (mmol / l) TAC (mmol / L) GSH (moll / L) MDA (moll / L)	Caesarean Need for insulin therapy after the intervention Pre eclampsia RN polyhydramniosMaternal hospitalization Premature birth Macrosomia> 4000 gr Gestational age (wk) Weight of the RN (g) RN height (cm) RN head cephalic (cm) Weight index (kg/m3) 1 min Apgar 5 min Apgar Hospitalization of the Newborn Newborn Hypoglycemia Mother’s weight (g) BMI (kg / m^2^)	Vitamin D (ng / ml) Calcium (mg / dl) FPG (mg / dl) Inulin (mIU / ml) HOME-IR HOMA-B QUICKI Total cholesterol (mg/dl) Triglycerides (mg/dL) LDL cholesterol (mg/dL) HDL Cholesterol (mg/dl) Total: HDL-cholesterol hs-CRP (ng / ml) TAC (mmol / L) GSH (mmol / L) Maternal health BMI(kg / m^2^)	Vitamin D Caesarean Need fo insulin therapy after the intervention Pre eclampsia RN polyhydramnios Maternal hospitalization Premature birth Macrosomia > 4000 gr Gestational age (wk) Weight of the RN (g) RN height (cm) RN head cephalic (cm) 1 min Apgar 5 min Apgar Newborn hospitalization—Newborn hypoglycemia Newborn hyperbilirubinemia	25-hydroxyvitamin D (25(OH)D) FPG levels serum insulin levels homeostasis model of assessment (HOMA) insulin resistance (HOMA-IR) HOMA of β cell function (HOMA-B)	fasting plasma glucose (FPG), insulin, vitamin D calcium levels

G1: Intervention, G2: Control, NI: Not Informed.

* American Diabetes Association guidelines: Screening for DMG using a one-step strategy -> performing the oral overload test with 75g of glucose (TTG75g), between 24–28 weeks of gestation in women not previously diagnosed with overt diabetes, evaluating the concentration of (overnight of 8 hours), one and two hours after the ingestion with measurement of plasma glucose. The diagnosis of DMG is performed when any of the plasma glucose values are reached or exceeded, with fasting: 92 mg / dL (5.1 mmol / L), 1 hour: 180 mg / dL (10.0 mmol / L) and 2 hours: 153 mg / dL (8.5 mmol / L).

** American Diabetes The Association guidelines: Tracking for DMG using two-step strategy -> 50g oral glucose overload test (TTG50g) between 24–28 weeks of gestation, not fasting, with plasma glucose measurement in 1 hour. After 1 hour if plasma glucose values ≥ 140 mg / dl (7.8 mmol / L), carry out an oral overload test with 100g of glucose (TTG100g), measured in fasting, 1 hour, 2 hours and 3 hours after glucose overload. The diagnosis of GDM is confirmed by altering at least two of the following four levels if reached or exceeded; Fasting: 95 mg / dL (5.3 mmol / L), 1 hour: 180 mg / dL (10.0 mmol / L), 2 hours: 155 mg / dL (8.6 mmol / L) and 3 hours: 140 mg / dL mmol / L).

### Risk of bias

**Fig 2:**
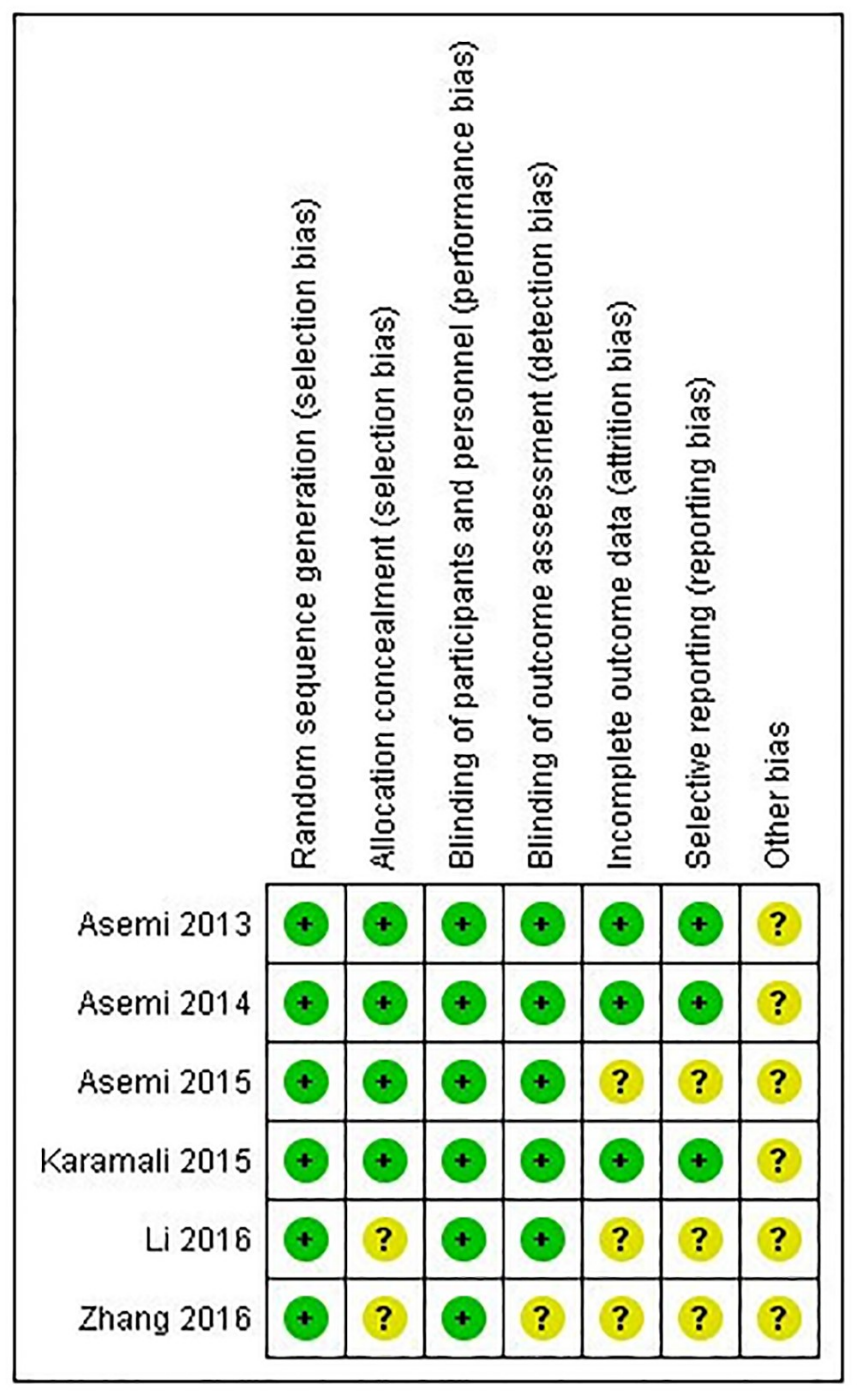
Assessment of bias risk of randomized clinical trials included.

The risk of bias assessment is shown in Fig 2. Regarding the randomization process, all trials used a random numerical list generated by a computer system, therefore being classified as low risk.

Regarding allocation concealment, in the four Iranian trials the allocations were concealed from the researcher and participants until the main analyses were completed (low risk of bias). Zhang et al. (2016) and Li et al. (2016) did not provide any information in regard to allocation process. Then, they were classified as at unclear risk of bias.

In all included trials there was blinding of participants and personnel. For blinding of outcome assessment, Zhang et al. (2016) did not provide any statement regarding this domain, and in the remaining trials the outcomes were analyzed in a blinded way.

Regarding incomplete outcome data, Asemi et al. (2013) et al. and Asemi et al. (2014) reported that patients who did not complete the treatment regimen were included in the final analysis (low risk). Karamali et al. (2015) reported that all participants completed the treatment (low risk). In the trial of Asemi et al. (2013), Zhang et al. (2016) and Li et al. (2016), less than 15% patients were lost to follow-up and were not included in the final analysis, although the number of patients who were lost was not significantly different between the groups, we considered risk of bias to be unclear.

In the three trials where the protocol was available (Asemi et al. (2013), Asemi et al. (2014) and Karamali et al. (2015)), all outcomes were reported as intended, and we considered low risk of bias for selective reporting. For the remaining included trials, there was not enough information to fully assess the potential for selective reporting bias, therefore they were judged as being an unclear risk.

### Meta-analysis

#### Maternal outcomes

Regarding our primary outcomes, none of the trials evaluated maternal adverse events (e.g., hypercalcemia, kidney stone, among others), neonatal death (within 28 days of delivery), stillbirth, low birth weight (< 2,500 g), or neonatal infection (respiratory infections within 28 days after delivery).

Two trials evaluated the frequency of preeclampsia and preterm newborns. [20, 21] Due to scarce number of events, effect estimates were imprecise and consequently differences between groups were not statistically different (Table 3).

**Table 3:** Summary of findings based on the GRADE Approach.

Outcomes	Patients (n°)	Risk of bias	Inconsistency	Indirectness	Imprecision	Publication Bias	Relative Risk (95% CI)	Quality of evidence
**Preeclampsia**	105 (2 studies)	Serious (-1) *	No	No	Very Serious (-2) **	Probably not	0.34 (0.04–3.17))	⊕○○○ very low^2^
**Preterm newborns**	105 (2 studies)	Serious (-1) *	No	No	Very Serious (-2) **	Probably not	1.02 (0.18–5.71)	⊕○○○ very low^2^
**Cesarean deliveries**	105 (2 studies)	Serious (-1) *	No	No	Very Serious (-2) **	Probably not	0.55 (0.26–1.16)	⊕○○○ very low^2^
**Maternal hospitalization**	105 (2 studies)	Serious (-1) *	No	No	Very Serious (-2) **	Probably not	0.13 (0.02–0.98)	⊕○○○ very low^2^
**Frequency of hospitalized newborns.**	105 (2 studies)	Serious (-1) *	No	No	Serious (-1) **	Probably not	0.40 (0.23–0.68)	⊕⊕○○ low^2^
**Polyhydramnios**	105 (2 studies)	Serious (-1) *	No	No	Very Serious (-2) **	Probably not	0.17 (0.03–0.89)	⊕○○○ very low^2^

Note: To determine a GRADE quality of the evidence, the GRADE approach begins by assigning findings to one of two starting levels of quality depending on the study design. Randomized trials are high quality, while observational studies are low quality. Additionally, two other levels exist; moderate and very low. This gives four levels: High, Moderate, Low and Very low. Studies can then be up-or downgraded based on certain factors:
a) Risk of bias (-1 if serious risk of bias, -2 if very serious risk of bias). b) Inconsistency or heterogeneity of evidence (-1 if serious inconsistency, -2 if very serious inconsistency) c) Indirectness of evidence (-1 if serious, -2 if very serious) d) Imprecision of results (-1 if wide confidence interval, -2 if very wide confidence interval) e) Publication bias (-1 if likely, -2 if very likely)

* Many domains classified as unclear.

** Small events and large confidence interval. Low Quality of Evidence: the authors are not confident in the effect estimate and the true value may be substantially different from it. Very Low Quality of Evidence: the authors do not have any confidence in the estimate and it is likely that the true value is substantially different

The meta-analysis of frequency of cesarean deliveries did not show significant differences between groups [20, 21], but did describe that cesarean deliveries tended to be lower in the interventional group (Table 3).

**Fig 3:**
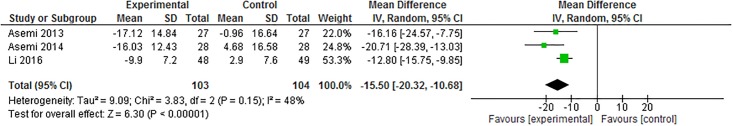
Meta-analysis of the decrease in fasting blood glucose at the end of the study.

The decrease in fasting blood glucose at the end of the trial was reported by Asemi et al. (2013), Asemi et al. (2014), and Li et al. (2016); the meta-analysis presented a significant difference between groups, favoring the supplemented group (MD: -15.50, 95% CI: -20.32 to -10.68) (Fig 3). In the trial by Zhang at al. (2016), there was no difference between groups for this outcome; however, data could not be plotted due missing standard deviation.

The outcome of need for maternal hospitalization, as measured by Karamali et al. (2015) and Asemi et al. (2015), showed a significant difference between groups, favoring vitamin D supplementation (RR: 0.13; 95% CI: 0.02 to 0.98, Table 3), but with an important imprecision due to wide confidence interval.

Asemi et al. (2013) and Asemi et al. (2014) reported the lipid profile variation, with difference observed favoring the intervention group for HDL increase (MD: 2.89, 95% CI: 0.78 to 5.00) and LDL reduction (MD: -13.68, 95% CI: -21.61 to -1.76).

HOMA-IR and HOMA-B were reported by Asemi et al. (2013) and Asemi et al. (2014) and showed a significant difference between groups, favoring the supplemented group in HOMA-IR (MD: -1.58, 95% CI: -2.19 to 0.97), and no difference in HOMA-B.

Regarding the change in maternal vitamin D concentration at the end of the four Iranian trials, a significant difference was found between the supplemented and control groups, with a higher concentration of 25(OH)D in the intervention group (MD: 18.82, 95% CI: 14.95 to 22.68, I2 = 0).

Asemi et al. (2013), Asemi et al. (2014), and Karamali et al. (2015) evaluated serum calcium concentrations at the end of the trials, no patients developed hypercalcemia.

Asemi et al. (2015) and Karamali et al. (2015) reported the change in maternal BMI and the frequency of insulin need after the intervention, and there was no difference between the groups (MD: -0.02, 95% CI: -0.21 to 0.18 and RR: 0.2, 95% CI: 0.02 to 1.69, respectively).

**Fig 4:**
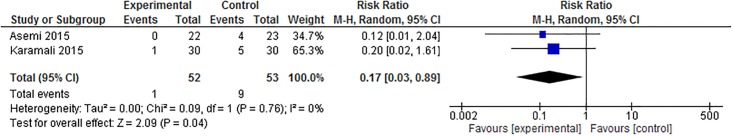
Meta- analysis of complications in the newborn (polyhydramnios).

Asemi et al. (2015) and Karamali et al. (2015) evaluated the frequency of polyhydramnios, the result was significantly lower in the intervention groups (RR: 0.17, 95% CI: 0.03 to 0.89; Fig 4).

#### Neonatal outcomes

The 5 minutes Apgar outcome was evaluated by Asemi et al. (2015) and Karamali et al. (2015). In Asemi et al. (2015), the Apgar score was higher in the supplementation group, and in Karamali et al. (2015), there was no difference between groups. The meta-analysis was not performed due to the presence of this heterogeneity between the two comparisons.

**Fig 5:**
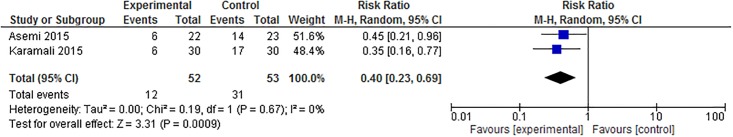
Meta Analysis of complications in the newborn (occurrence of hyperbilirubinemia).

Asemi et al. (2015) and Karamali et al. (2015) evaluated the frequency of hyperbilirubinemia. The frequency of newborns with this outcome was significantly lower in the intervention groups (RR: 0.40, 95% CI: 0.23 to 0.68; Fig 5).

**Fig 6:**
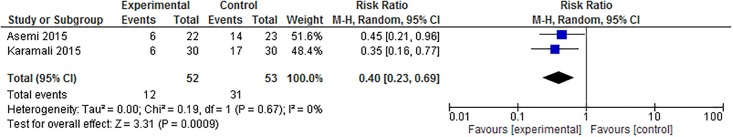
Meta-analysis of the frequency of hospitalized newborns.

The meta-analysis of frequency of hospitalized newborns, measured by Asemi et al. (2015) and Karamali et al. (2015), showed a significant difference between groups, favoring the vitamin D supplementation group (RR: 0.40, 95% CI: 0.23 to 0.69) (Fig 6).

Asemi et al. (2015) and Karamali et al. (2015) reported the frequency of macrosomic newborns. There was no difference between the supplemented and control groups (RR: 0.20, 95% CI: 0.04 to 1.13).

### Evaluation of quality of evidence according to the GRADE (Table 3)

The quality of evidence assessment was performed for the outcomes that were thought to be most important from a patients’ point of view and were plotted in the meta-analysis. The quality of evidence was very low for the frequency of preeclampsia, prematurity, caesarian deliveries, and maternal hospitalization. For these outcomes, it was necessary to rate them down one level for risk of bias and two levels for imprecision. For newborn hospitalization the quality evidence was low; it was necessary to rate it down one level of evidence for risk of bias and imprecision. As less than ten RCTs were included in this review, we could not analyze the presence of publication bias.

## Discussion

During pregnancy, maternal vitamin D deficiency has been associated with adverse maternal and fetal outcomes, such as increased incidence of preeclampsia, insulin resistance, diabetes mellitus, and increased frequency of cesarean delivery [12, 13, 28, 29].

As vitamin D deficiency has been a common and often underdiagnosed health problem worldwide, and considering the benefits of vitamin D supplementation in GDM control [14] and on maternal and neonatal outcomes in normoglycemic pregnant women [30], we performed this systematic review and meta-analysis to evaluate the efficacy of vitamin D supplementation in pregnant women with GDM. Our hypothesis was that this supplementation would protect pregnant women against maternal and neonatal complications related to GDM.

Six trials fulfilled our eligibility criteria and were included in this review [14, 18–22]. Four hundred and fifty-four pregnant women with GDM were randomized to supplementation with vitamin D_3_ or placebo. The data of five trials could be plotted in the meta-analysis. Regarding maternal risk in GDM, the most important outcomes evaluated were frequency of preeclampsia, hospitalization and cesarean delivery. None of these comparisons showed significant differences between groups; however, due to the small number of events and trials, the 95% CI was very wide, resulting in a very low quality of evidence according to GRADE approach.

The most important neonatal outcomes that could be plotted on meta-analysis were frequency of prematurity, need for hospitalization of newborn. For prematurity, there was no difference between the groups, but with a very wide CI of 95%. The meta-analysis showed that offspring of women with GDM and without vitamin D supplementation were more likely to require hospitalization, presenting a risk difference in favor of the intervention of 30%. However, the quality of evidence was low, resulting in a lack of confidence in the effect estimate and that the true value may be substantially different.

Our systematic review had some limitations, with the main one being related to the small number of trials and patients included. All were single center trials, which tend to provide larger treatment effects than do multicenter RCTs, and hence, they should be carefully used in decision making [31]. Additionally, no trial evaluated adverse events of vitamin D supplementation. Another important limitation is we combined data from two trials testing supplementation with vitamin D alone and two with vitamin D plus calcium. Although there was no heterogeneity in the four outcomes which differences between groups favored vitamin D supplementation (Figs 3 to 6), this may introduce confounding effects since the interaction between the two supplements and any independent effects are not clear.

In the literature, this is the first published systematic review evaluating the effects of vitamin D supplementation focused on pregnant women with GDM. In the Cochrane Database of Systematic Reviews, there is a review published in 2016 that evaluated vitamin D supplementation in normoglycemic pregnant women. [30] Data from two RCTs (219 pregnant women) showed that women with supplementation of vitamin D alone, compared to placebo, had a lower risk of preeclampsia, similar risk of GDM; however, these had low and very low qualities of evidence, respectively. Regarding infant outcomes, supplementation compared to no intervention or placebo (three RCTs with 477 women) suggested that supplementation reduces the risk of preterm births (8.9% versus 15.5%; RR 0.36, 95% CI: 0.14–0.93) and a birthweight below 2500 g (RR 0.40, 95% CI: 0.24–0.67). Despite a moderate quality of evidence for these two last outcomes, the authors concluded that is unclear whether vitamin D supplementation in normoglycemic pregnancy improves maternal and neonatal outcomes. The most recent systematic review including pregnant women with vitamin D supplementation is from Roth et al. [32] published in 2017. From 43 eligible trials (8406 participants), maternal clinical outcomes were rarely reported, and available data did not provide evidence of benefit. Overall, vitamin D increased mean birth weight of 58.33 g (95% CI: 18.88 g to 97.78 g) and reduced the risk of small for gestational age births (RR 0.60, 95% Cl: 0.40 to 0.90), but findings were not robust in sensitivity and subgroup analyses.

## Conclusions

### Implications for practice

We did not find moderate or high quality evidence from RCTs suggesting that in pregnant women with GDM, vitamin D supplementation, when compared with placebo, improves adverse maternal and neonatal outcomes related to GDM.

### Implications for research

Further large, well done, and multicenter RCTs are required to determine the efficacy and safety of vitamin D supplementation in pregnant women with GDM, with the aim to improve the maternal and neonatal adverse outcomes related to GDM.

## Supporting information

S1 Appendix Search strategy. (DOCX)
