# Supplementary material for: Efficacy of vitamin D supplementation in gestational diabetes mellitus: Systematic review and meta-analysis of randomized trials
Source: PLoS One. 2019 Mar 22;14(3):e0213006. doi: 10.1371/journal.pone.0213006 (PMC6430411; doi:10.1371/journal.pone.0213006)
Supplement: S1 Appendix — (DOCX) [file pone.0213006.s001.docx]

**APPENDIX 1 - MOTHER SEARCH STRATEGY**

PUBMED

#1 "Diabetes, Gestational"[Mesh] OR (Diabetes, Pregnancy-Induced) OR (Diabetes, Pregnancy Induced) OR (Pregnancy-Induced Diabetes) OR (Gestational Diabetes) OR (Diabetes Mellitus, Gestational) OR (Gestational Diabetes Mellitus) OR (GDM)

#2 "Diabetes Mellitus, Type 2"[Mesh] OR (NIDDM) OR (Maturity-Onset Diabetes) OR (Diabetes Mellitus, Noninsulin-Dependent) OR (Diabetes Mellitus, Adult-Onset) OR (Adult-Onset Diabetes Mellitus) OR (Diabetes Mellitus, Adult Onset) OR (Diabetes Mellitus, Ketosis-Resistant) OR (Diabetes Mellitus, Ketosis Resistant) OR (Ketosis- Resistant Diabetes Mellitus) OR (Diabetes Mellitus, Maturity-Onset) OR (Diabetes Mellitus, Maturity Onset) OR (Diabetes Mellitus, Non Insulin Dependent) OR (Diabetes Mellitus, Non-Insulin-Dependent) OR (Non-Insulin-Dependent Diabetes Mellitus) OR (Diabetes Mellitus, Noninsulin Dependent) OR (Diabetes Mellitus, Slow-Onset) OR (Diabetes Mellitus, Slow Onset) OR (Slow-Onset Diabetes Mellitus) OR (Diabetes Mellitus, Stable) OR (Stable Diabetes Mellitus) OR (Diabetes Mellitus, Type II) OR (Maturity-Onset Diabetes Mellitus) OR (Maturity Onset Diabetes Mellitus) OR (MODY) OR (Type 2 Diabetes Mellitus) OR (Noninsulin-Dependent Diabetes Mellitus)

#3 "Pregnancy"[Mesh] OR (Pregnancies) OR (Gestation)

#4 "Vitamin D"[Mesh] OR (D04.808.812.768) OR (D014807) OR (1406-16-2) OR (Vitamin D3) OR (25(OH)D) OR (25-hydroxyvitamin D)

#5 "Vitamin D Deficiency"[Mesh] OR (Deficiency, Vitamin D) OR (Deficiencies, Vitamin D) OR (Vitamin D Deficiencies) OR (Vitamin D supplementation) OR (Vitamin D Insufficiency)

#6 "Cholecalciferol"[Mesh] OR (Calciol) OR ((3 beta,5Z,7E)-9,10-Secocholesta- 5,7,10(19)-trien-3-ol) OR (Vitamin D 3) OR (Vitamin D3) OR (Cholecalciferols) OR (D04.808.247.222.159) OR (D04.808.247.808.146) OR (D04.808.812.768.196) OR (D10.570.938.146) OR CD002762) OR (1C6V77QF41)

#1 OR (#2 AND #3) AND (#4 OR #5 OR #6) = 239
